# Assessing the Cost of Nutritionally Adequate and Low-Climate Impact Diets in Finland

**DOI:** 10.1016/j.cdnut.2024.102151

**Published:** 2024-04-03

**Authors:** Xavier Irz, Laura Sares-Jäske, Heli Tapanainen, Jyrki Niemi, Laura Paalanen, Merja Saarinen, Liisa M Valsta

**Affiliations:** 1 Department of Economics and Management, University of Helsinki, Helsinki, Finland; 2 Natural Resources Institute Finland, Bioeconomy Policies and Markets Group, Helsinki, Finland; 3 Finnish Institute for Health and Welfare, Department of Public Health and Welfare, Helsinki, Finland; 4 Natural Resources Institute Finland, Sustainability Science and Indicators Group, Helsinki, Finland

**Keywords:** diet, food consumption, optimization, sustainability, environmental impact, just transition, cost, climate change

## Abstract

**Background:**

Dietary changes form an important component of the sustainability transition of food systems but could be hindered by the cost of sustainable diets.

**Objectives:**

This study aimed to characterize the cost of nutritionally adequate and culturally acceptable diets with low-greenhouse gas emissions (GHGEs) in Finland.

**Methods:**

Two optimization models were built to find diets complying with nutritional and emissions requirements. The first model minimizes diet cost and the second one deviation from current diets. Both are calibrated to Finnish sociodemographic groups using dietary intake data, household budget survey data (for prices), and life cycle assessment coefficients (for GHGE). Three scenarios are simulated: “Health only” imposes only compliance with nutritional constraints, whereas “Health and GHGE-33%” and “Health and GHGE-50%” impose, in addition, minimum reductions in GHGE.

**Results:**

Minimum cost diets have a low-carbon footprint [−65% (−73%) for females (males)] and low cost [−69% (−73%) for females (males)] when compared with current diets but lack diversity and cultural acceptability. The more culturally acceptable health-only minimum deviation diets are marginally less costly and have a lower climate impact than baseline diets across all population groups. Reducing GHGE results in a substantial decrease in the cost of the minimum deviation diets. The lower cost of the minimum deviation diets with reduced GHGE results from both intercategory and intracategory substitutions.

**Conclusions:**

Affordability is not the key obstacle to the adoption of nutritionally adequate and lower GHGE diets, but cultural acceptability is. Reducing the climate footprint of diets can generate side benefits in terms of nutrition and affordability, which confirms that dietary change should be central to the sustainability transition of the Finnish food system. However, more attention should be paid to the issues of taste, convenience, social norms, and other aspects determining the cultural acceptability of sustainable diets.

## Introduction

Recent research has produced a strong scientific consensus that the global food system is fundamentally unsustainable [1,2] and that population-level dietary changes should form a central component of its required transformation toward sustainability [[3], [4], [5]]. This consensus has emerged primarily from the evidence that, given the characteristics of foods in the prevailing food system, nutritional adequacy is compatible with a large decrease in dietary greenhouse gas emissions (GHGEs). This has been demonstrated globally by the EAT-Lancet commission [4] and in various countries (e.g., Australia [6], Finland [7,8]), both based on optimization models [9] and the analysis of diets proposed by experts [10,11] or observed in current populations [12,13]. Other recently published results suggest that the synergies between nutritional and climate outcomes extend to other domains of environmental sustainability such as biodiversity preservation [[14], [15], [16]], nutrient loading [17], land use, blue water consumption, or ecosystem status [18], although trade-offs have also been found [19].

Albeit insightful, this body of research remains limited in its power to inform policy making for a sustainable dietary transition because of its relative neglect of the third socioeconomic and cultural dimension of the sustainability tripod. The importance of aspects other than health and the environment in sustainable diets is in fact recognized in the most widely accepted definition of those diets as follows: the “dietary patterns that promote all dimensions of individuals’ health and wellbeing; have low environmental pressure and impact; are accessible, affordable, safe and equitable; and are culturally acceptable” [2]. However, in practice, the analyses of sustainable diets taking into account those elements remain relatively rare, probably because of the difficulty of bringing largely qualitative notions such as “cultural acceptability” into the quantitative models commonly used to investigate the sustainability properties of diets. As an example, even the ambitious, scientifically rigorous and widely publicized EAT-Lancet report has been criticized for its near exclusive focus on health and the environment, at the expense of an analysis of the potential cost and economic feasibility of the advocated dietary changes [20]. That economic analysis was carried out only independently by other researchers [21] after publication of the EAT-Lancet study, showing that “the EAT-Lancet diet was not actually affordable by many of the world’s poor” [21]. In a European context, a few studies have analyzed the cost of current and relatively more sustainable diets, such as the Mediterranean diet in Italy [22], but we note the absence of any research for our area of interest, namely Northern European countries such as Finland.

The lack of consideration of socioeconomic and cultural issues in general, and affordability in particular, in the analysis of sustainable diets represents a serious shortcoming and important research gap. Devising policies aiming at change toward relatively unaffordable diets is unlikely to be effective, fair, or politically feasible. Yet, the evidence to date is too limited to be able to claim that the diets often recommended by governments or environmental nongovernmental organizations do not impose prohibitive costs on consumers, and some published results invite caution, as explained next.

At a global level, it was recently estimated by the FAO that, in 2021, a large share of world population (42%, or 3.1 billion people) was simply unable to afford a healthy diet, costing an average of 3.66 purchasing power parity dollars worldwide [23]. More specifically related to high-income countries, there is also a large body of evidence showing that the nutritional quality of diets is often positively related to their cost because energy-dense but nutrient-poor foods rich in added sugar and fat tend to be cheaper per kilocalorie than nutrient-rich foods such as fruits and vegetables [[24], [25], [26]]. It follows that food costs pose a significant barrier for many consumers attempting to balance good nutrition with a budget constraint [27]. As it is well established that the economic importance of food in consumers’ budgets is inversely related to income—a regularity known as Engle's law in economics [28]—it follows that this barrier may be particularly pronounced for the least well-off in society, even in high-income countries. Thus, care must be taken to explicitly address the potential criticism that policies promoting the transition toward sustainable diets may be unfair.

More optimistically, previous work on diets with lower environmental impact has robustly showed the need to reduce consumption of animal products and increase that of plant-based foods. As at least some unprocessed plant-based foods are cheaper than many meats and dairy products, this opens the enticing possibility that the synergies between health and climate/environment may also generate budgetary savings for the consumer, although we also acknowledge that highly processed meat substitutes often cost more than conventional meat [29]. Nevertheless, existing literature to date only allows us to state that the affordability of sustainable diets remains an open empirical question that deserves more attention, as stated in conclusion of a recent systematic review of the field [30]. The authors of that review reported that, from 24 studies published between 2017 and 2023 and included in their analysis, only 7 had reported any information about the cost of diets.

Against this background, this article investigated the cost of nutritionally adequate diets with a lower climate impact than current diets (henceforth referred to as “reduced GHGE diets” for convenience) while taking the issue of cultural acceptability into account in the analysis. We investigated several specific research questions, such as follows, in the context of the Finnish adult population: *1*) What is the minimum cost of a reduced GHGE and nutritionally adequate diet, and is this diet likely to be culturally acceptable? *2*) How costly are reduced GHGE, nutritionally adequate and culturally acceptable diets? *3*) Does the magnitude of the cost barrier to the adoption of sustainable diets vary across sociodemographic groups? The analyses have been conducted separately for males and females because, based on previous studies, diet quality differs between the 2 sexes [31,32].

## Methods

### Data

#### Dietary intakes and food composition

The National FinDiet 2017 Survey [33] provided a detailed description of the average diet of the Finnish adult population differentiated by sex, income quintile, and educational level. The nationally representative FinDiet 2017 Survey is a subsample (*n* = 3099) of the FinHealth 2017 Study (*N* = 10247) [34]. Our analysis used data on 1655 adults aged 18‒74 y (875 females and 780 males, 53% of the invited) with 2 nonconsecutive 24-h dietary recalls. Table 1 provides the distribution across age, education level, and income level. The in-house dietary software Finessi [Finnish Institute for Health and Welfare (THL), Finland] and the National Food Composition Database Fineli (FCDB; Release 20; open-access version available online: https://fineli.fi/fineli/en/index?) were used to calculate the nutrient intakes of different diets. Food consumption was estimated at ingredient level after disaggregating the consumed foods according to the recipes of the FCDB. The nutrient composition of a food category was derived by calculating weighted sum of nutrient intakes of all food items belonging to the food category. The weights for every food item were calculated as the share of the consumption of a food item from the consumption of the whole food category in the FinDiet 2017 Survey data. The models described in the following section were built on a food categorization incorporated in the FCDB. Some categories were aggregated for this analysis, but the final classification (74 food categories) elaborated by nutritionists was kept sufficiently disaggregated to allow for precise nutritional and climate impact assessments. In some cases, these 74 food categories were aggregated after completion of the optimization process into 16 main food categories to facilitate reporting and analysis. (We keep this terminology of food categories for the more disaggregated categorization of 74 food ingredients and “main food categories” for the more aggregated categorization made of 16 categories.)

**TABLE 1:** The participants of the FinDiet 2017 Survey by sex, age group, education tertile, and income quintile.

	Men		Women	
	*n*	%	*n*	%
Age group (y)				
18–24	47	6	52	6
25–44	221	28	259	30
45–64	308	39	317	36
65–74	204	26	247	28
Education				
Low	259	33	269	31
Medium	258	33	305	35
High	256	33	285	33
Missing	7	1	16	2
Income				
Quintile 1	156	20	158	18
Quintile 2	173	22	132	15
Quintile 3	147	19	221	25
Quintile 4	132	17	157	18
Quintile 5	156	20	173	20
Missing	16	2	34	4
Total	780	100	875	100

#### Background information and sociodemographic groups

The total self-reported years of education, starting from primary school and including all fulltime education, were categorized into tertiles (low, medium, and high) according to sex and birth year. The income quintile was based on questions on total household income during the previous year before tax deductions and on the number of adult and underage household members. Total household income was divided by a weighted sum of household members, assigning a weight of 1.0 to the first adult, 0.7 to additional adults, and 0.5 to underage household members, in line with the equivalence scale of the Organization for Economic Cooperation and Development (OECD) [35]. The resulting values were categorized into sex-specific quintiles. The groups included in the analysis for each sex were as follows: whole adult population; all 3 educational tertiles, henceforth referred to as the low education, medium education, and high-education groups; and 3 income quintiles, referred to as income quintile 1, income quintile 3, and income quintile 5 groups.

#### Environmental coefficients

The GHGE coefficients were generated using the life cycle assessment climate impact coefficients recently published by Saarinen et al. [8]. The coefficients are reported in the supplemental material of that article as “Data sheet 1.”

#### Prices

Data on food and drink prices at a sufficient level of product disaggregation were not directly available and had to be constructed from the 2016 round of Statistic Finland’s Household Budget Survey (https://stat.fi/en/statistics/ktutk; accessed 10 November, 2023; 2016 is the year closest in time to that of the collection of the dietary intake data). The survey gives a detailed description of each respondent household’s demographic and social structure, sources of revenue, use of money, and purchase of foods for consumption at home. The food data provide the expenditure value and physical quantities for 259 foods and nonalcoholic beverages grouped according to the Classification of Individual Consumption by Purpose (COICOP). The data were recorded by each participating household (*n* = 3673) in a diary over a 2-wk period and backed up by actual sales receipts. Prices for the 259 COICOP food and drink categories were calculated by the ratio of average expenditure to physical quantity for the whole sample. The COICOP categories were then matched whenever possible to the 74 FinDiet food categories, and the price of each of those categories was calculated as the weighted average of the matched COICOP categories.

### The diet optimization models

#### The objective functions

We built 2 simulation models that differ solely in their objective functions. The minimum cost model was constructed by minimizing the cost of the diet subject to linear constraints described in detail further. This defines a linear optimization problem that is easily solved for a global solution using standard algorithms and packages (in our case, the R package lpSolve) [36].

The minimum deviation model was constructed by minimizing the sum of squared relative deviations from the observed average diet, subject to a set of linear constraints, also described further. Formally, the objective function is $\underset{x}{\mathrm{Min}}\sum_{i=1}^{n}{\left(\frac{x_{i}-x_{i}^{0}}{x_{i}^{0}}\right)}^{2}$, where *x* denotes a *n*-vector of average consumption *x*_*i*_ of each food *i*, and $x_{i}^{0}$ defines the observed (=current) average consumption of food *i* in the population of interest. The procedure limits departure from the observed average diet and, by doing so, preserves the cultural acceptability and realism of the simulated dietary changes. As noted in a recent scoping review of indicators for sustainable healthy diets [37], the approach of ensuring cultural acceptability by minimizing departure from the current diet is the most frequently used in the sustainable diet literature. It relies on the idea that observed diets already embed consumer preferences and the difficult but stable trade-offs involved in food choices. Hence, radical changes from observed choices are unrealistic [38]. This general line of reasoning has been used previously in many published studies on diet optimization [[38], [39], [40], [41], [42]]. In addition, the food habit constraints described further also contribute to ensuring the cultural acceptability of the simulated minimum deviation diets.

The abovementioned structure defines a classic quadratic programming problem, in which a quadratic objective function is minimized subject to a set of linear equality and inequality constraints. Although the numeric solutions to those types of problems can be local rather than global, the exact form of our objective function ensures that this is not an issue in this study because the matrix defining the quadratic term of the objective function is always symmetric positive definite. This makes the quadratic programming problem strictly convex, and such problems have a single global minimum [43], as noticed elsewhere in the diet optimization literature [44]. Thus, the numeric optimization derived by applying the R package quadprog [45] gives the global solution to the minimum deviation diet optimization problem.

#### The nutritional constraints

A first linear constraint imposes the constancy of energy intake, which is set at its observed level in the dietary intake data. Thus, all simulations are isocaloric, and we abstract from addressing the relevant but different issue of optimal energy intake in order to focus solely on that of diet composition.

A set of constraints defines the minimum for recommended [46,47] or safe [48] daily intake and the maximum for recommended daily intake or upper level for safe intake for a detailed list of macronutrients (*n* = 30), vitamins (*n* = 13), and minerals (*n* = 18) listed in Supplemental Table 1. The values were drawn from the Nordic Nutrition Recommendations 2012 [46] and Finnish Nutrition Recommendations 2014 [49] and for amino acids from the WHO protein and amino acid recommendation [48], namely individual amino acid requirement with added 24% safety margin. This was a slightly more conservative approach than using the average requirement reference values. This approach was chosen due to the fact that the used data in this study did not represent usual intake of the population groups but were group averages and thus did not fulfill the prerequisites for using the average requirement values as a reference. There was, however, an exception in using the recommended daily intake type of reference value for the constraints. The minimum iron intake for females was set to its concentration observed in the current Finnish diet that meets the recommended daily intake of postmenopausal women but only the average iron requirement in case of premenopausal women [32,46].

The detailed list of recommended or safe daily intakes makes clear that the adequacy of intakes of macronutrients (proteins, fatty acids, and carbohydrates) and micronutrients (vitamins and minerals) is explicitly taken into account in the analysis. Imposition of those constraints ensures that all solution diets are, by construction, nutritionally adequate according to the selected set of nutritional criteria.

#### The climate impact constraint

A single environmental constraint imposes an exogenously given maximum level of GHGE from the diet, set at two-thirds of its current level (health and GHGE -33% scenario) or half of its current level (health and GHGE -50% scenario). In addition, a “health-only” scenario imposes only the nutritional constraints and lets the level of GHGE be determined by the model without any restriction.

#### The food system constraint

A constraint is introduced to reflect the jointness of dairy and beef production in the Finnish food system [50]. At present, the beef-to-dairy ratio cannot realistically fall under a minimum level as ∼80% of beef in Finland originates from the dairy chain. The study of the Dutch diet by Broekema et al. [51] introduced a similar constraint. We estimated that, from the Finnish dairy chain, for each gram of beef carcass, 33.9 g of raw milk are produced. The beef content of the relevant food ingredients (in parentheses) was also estimated to quantify the ratio of raw milk to beef production: beef (100%), offals (88%), meat products (50%), sausages (7.5%), sausage cuts (7.5%), and meat cuts (7.5%).

#### The food habit constraints (minimum deviation model only)

The minimum deviation model aims at maximizing the cultural acceptability of the simulated diets. This is partly achieved by the choice of the objective function, which minimizes overall deviation from observed diets. However, this is reinforced at the level of each food category by the imposition of food habit constraints ensuring that, for any particular category, the simulated intake is not extremely unusual in view of the distribution observed in the FinDiet sample. Formally, we impose that the optimal consumption of any food category should be no less than the 10th centile of the consumption distribution of that food in the population of interest and no more than the 90th centile, following the assumptions of Vieux et al. [9].

## Results

### How costly are nutritionally adequate and reduced GHGE diets?

Our analysis starts with the examination of the minimum cost diets produced by the minimum cost model, as reported in Table 2 for an average adult male and Supplemental Table 2 for an average adult female. By construction, those diets meet all nutritional requirements, and the last rows of the tables indicate that they have also both low cost and climate impact when compared with current diets. The cost of the minimum cost diets is only a fraction of that of the current average diet (31% for a female and 27% for a male). Further, the GHGE of the minimum cost diets are also only a small percentage of their current levels (35% for a female and 27% for a male), so that the GHGE constraint imposed by the model in some scenarios (e.g., health and GHGE -50%) is never binding, which means that the model would choose those low-GHGE diets even in the absence of any GHGE constraint. However, the minimum cost diets severely lack diversity as, for both genders, they are composed of only 12 of 74 foods in our classification. The bulk of the diet originates from cereals (mainly wheat and rye), potatoes, as well as liquid milk (skimmed and high fat). Fruits are not consumed, whereas consumption of vegetables decreases considerably compared with current diets in terms of both quantity and variety (only cabbage is consumed). The consumption of fish remains at its current level for females and decreases slightly for males. Consumption of meat drops to very small levels and is exclusively composed of offal. Oil and margarine complete those rudimentary diets.

**TABLE 2:** The minimum cost diet for an average adult male.

Main food category	Quantity (g/cap/d)		GHGE (gCO_2_e/cap/d)		Cost (€/cap/d)	
	Current diet	Minimum cost diet	Current diet	Minimum cost diet	Current diet	Minimum cost diet
Alcohol	146.2	0.0	0.2	0.0	0.83	0.00
Beer and cider	127.0	0.0	0.2	0.0	0.61	0.00
Wine and spirit	19.2	0.0	0.0	0.0	0.23	0.00
Beverages	680.0	0.0	0.2	0.0	0.40	0.00
Coffee	495.0	0.0	0.2	0.0	0.22	0.00
Soft drink	103.8	0.0	0.0	0.0	0.17	0.00
Tea	81.1	0.0	0.0	0.0	0.01	0.00
Cereals	157.5	358.7	0.2	0.5	0.25	0.42
Barley and barley products	1.1	0.0	0.0	0.0	0.00	0.00
Cereal/seed/soy drinks	7.7	0.0	0.0	0.0	0.03	0.00
Other grains	2.2	0.0	0.0	0.0	0.01	0.00
Oat and oat products	15.5	0.0	0.0	0.0	0.03	0.00
Rice	10.3	0.0	0.0	0.0	0.02	0.00
Rye	40.1	113.1	0.0	0.1	0.08	0.23
Starches	2.3	0.0	0.0	0.0	0.01	0.00
Wheat	78.4	245.6	0.1	0.3	0.06	0.19
Diet products	1.7	0.0	0.0	0.0	0.06	0.00
Sport foods, nutritional support	1.7	0.0	0.0	0.0	0.06	0.00
Eggs	24.2	0.3	0.1	0.0	0.08	0.00
Fats	52.8	45.7	0.3	0.2	0.28	0.22
Butter	4.9	0.0	0.1	0.0	0.02	0.00
Blended spread	13.6	0.0	0.1	0.0	0.08	0.00
Cooking and animal fat	5.1	0.0	0.0	0.0	0.02	0.00
Oils	10.3	24.9	0.0	0.1	0.06	0.14
Salad dressings	4.7	0.0	0.0	0.0	0.04	0.00
Margarines ≥ 55%	9.0	20.9	0.0	0.1	0.04	0.09
Margarines < 55%	5.1	0.0	0.0	0.0	0.02	0.00
Fish	35.7	19.3	0.1	0.1	0.45	0.20
Fish, seafood	25.4	19.3	0.1	0.1	0.27	0.20
Fish products	10.3	0.0	0.0	0.0	0.18	0.00
Flavoring	9.4	0.0	0.0	0.0	0.04	0.00
Condiments	9.4	0.0	0.0	0.0	0.04	0.00
Dried spices and herbs	0.1	0.0	0.0	0.0	0.00	0.00
Fruits	260.6	0.0	0.2	0.0	0.73	0.00
Malaceous fruit	32.0	0.0	0.0	0.0	0.06	0.00
Berries	24.2	0.0	0.0	0.0	0.09	0.00
Citrus fruit	23.9	0.0	0.0	0.0	0.04	0.00
Juice drink	75.8	0.0	0.1	0.0	0.32	0.00
Canned fruit	4.3	0.0	0.0	0.0	0.02	0.00
Other fruits	50.7	0.0	0.1	0.0	0.12	0.00
Juices, including vegetable juices	49.7	0.0	0.1	0.0	0.08	0.00
Ingredients	10.8	1.5	0.0	0.0	0.05	0.00
Miscellaneous ingredients	6.6	0.0	0.0	0.0	0.04	0.00
Salt	4.2	1.5	0.0	0.0	0.01	0.00
Sweeteners	0.0	0.0	0.0	0.0	0.00	0.00
Legumes and nuts	19.0	0.0	0.0	0.0	0.14	0.00
Nuts and seeds	6.7	0.0	0.0	0.0	0.09	0.00
Pulse vegetables and products	9.6	0.0	0.0	0.0	0.04	0.00
Soya products	2.7	0.0	0.0	0.0	0.01	0.00
Meat	181.1	12.3	2.4	0.2	1.44	0.10
Beef and lamb/mutton	35.6	0.0	1.1	0.0	0.37	0.00
Cold cuts, meat products	24.1	0.0	0.4	0.0	0.26	0.00
Offal	2.4	12.3	0.0	0.2	0.02	0.10
Pork and game	40.9	0.0	0.3	0.0	0.34	0.00
Poultry	42.8	0.0	0.2	0.0	0.20	0.00
Sausages	28.1	0.0	0.2	0.0	0.17	0.00
Cold cuts, sausages	7.3	0.0	0.1	0.0	0.07	0.00
Dairy	478.0	522.8	1.0	0.5	1.23	0.46
Ripened/processed cheese >17%	26.9	0.0	0.4	0.0	0.24	0.00
Ripened/processed cheese ≤17%	6.5	0.0	0.1	0.0	0.06	0.00
Unripened/fresh cheese >15%	4.1	0.0	0.0	0.0	0.04	0.00
Unripened/fresh cheese ≤15%	6.9	0.0	0.0	0.0	0.06	0.00
Cream	16.2	0.0	0.0	0.0	0.08	0.00
Quark	20.9	0.0	0.0	0.0	0.18	0.00
Ice cream	9.8	0.0	0.0	0.0	0.05	0.00
Skimmed milk	102.7	117.8	0.1	0.1	0.11	0.13
Milk with >2% fat	25.4	405.0	0.0	0.4	0.02	0.34
Milk powders	0.6	0.0	0.0	0.0	0.01	0.00
Milk with ≤2% fat	174.4	0.0	0.2	0.0	0.20	0.00
Soured/cultured milk	29.5	0.0	0.0	0.0	0.05	0.00
Fermented milk products, other	3.7	0.0	0.0	0.0	0.02	0.00
Yogurt	50.5	0.0	0.1	0.0	0.12	0.00
Potatoes	84.6	394.8	0.0	0.0	0.11	0.33
Potato products	12.9	0.0	0.0	0.0	0.05	0.00
Potato	71.7	394.8	0.0	0.0	0.06	0.33
Sugar	32.2	0.0	0.1	0.0	0.24	0.00
Chocolate	6.6	0.0	0.0	0.0	0.10	0.00
Jam	5.0	0.0	0.0	0.0	0.03	0.00
Sugar and syrups	14.4	0.0	0.0	0.0	0.03	0.00
Nonchocolate confectionery	6.2	0.0	0.0	0.0	0.09	0.00
Vegetables	176.6	59.2	0.3	0.0	0.57	0.14
Cabbage	13.6	59.2	0.0	0.0	0.03	0.14
Edible fungi	3.6	0.0	0.0	0.0	0.02	0.00
Root vegetables and tubers	28.3	0.0	0.0	0.0	0.06	0.00
Canned vegetables	19.0	0.0	0.0	0.0	0.08	0.00
Fruit vegetables	75.8	0.0	0.2	0.0	0.20	0.00
Leaf vegetables	21.6	0.0	0.0	0.0	0.17	0.00
Onion-family vegetables	14.7	0.0	0.0	0.0	0.02	0.00
All	2350.4	1414.8	5296.5	1452.1	6.90	1.88

Abbreviation: GHGE, greenhouse gas emission.

### How costly are nutritionally adequate, reduced GHGE, and relatively culturally acceptable diets?

**FIGURE 1:**
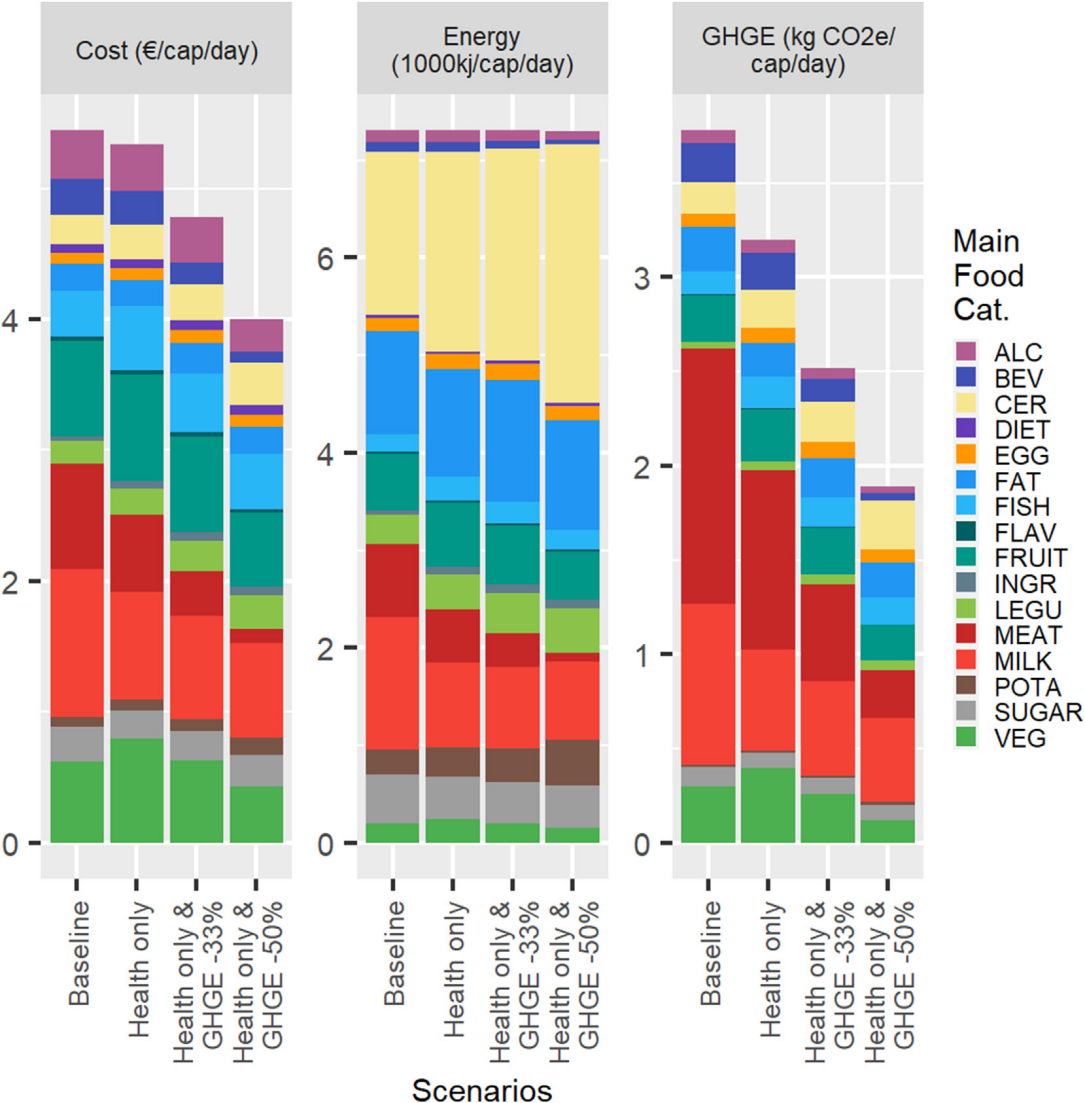
Cost, energy, and GHGE for the baseline and simulated minimum deviation diets: average adult female. The main food categories are defined in Table 2. GHGE, greenhouse gas emission.

**FIGURE 2:**
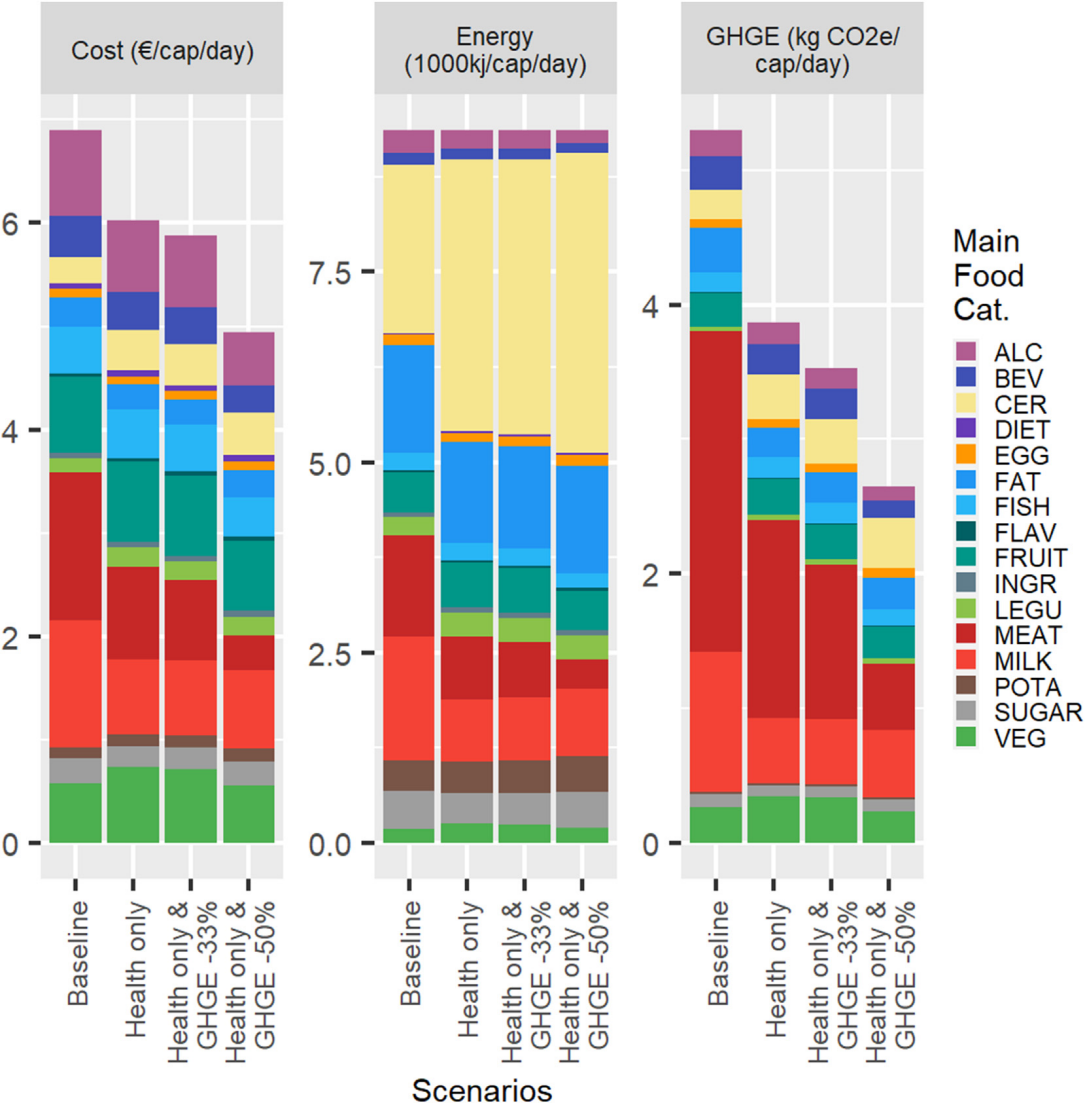
Cost, energy, and GHGE for the baseline and simulated minimum deviation diets: average adult male. The main food categories are defined in Table 2. GHGE, greenhouse gas emission.

The results of the simulations of the minimum deviation model are presented graphically in terms of diet costs, GHGE, and energy in Figure 1 for an average female and in Figure 2 for an average male, with the corresponding figures reported in TABLE 3, TABLE 4, TABLE 5 for an average female and Supplemental Tables 3–5 for an average male. For both sexes, the imposition of the nutritional constraints results in a small decline in diet cost (−2% for a female and −13% for a male), meaning that nutritionally adequate and culturally acceptable diets according to the minimum deviation model are slightly less costly than current diets. Further, we note in the right quadrants of FIGURE 1, FIGURE 2 that the nutritionally adequate (health only) diets also have significantly smaller GHGE than current diets (−27% for an average male compared with −15% for a female). When imposing larger reductions in GHGE of 33% or 50%, the simulated nutritionally adequate minimum deviation diets have even lower costs. For instance, the costs of the health and GHGE -50% minimum deviation diets are 28% and 27% less than those of current diets for an average male and female, respectively. Hence, we find strong synergies, rather than trade-offs, between nutritional adequacy, cost and GHGE reduction of the minimum deviation diets that preserve some aspect of cultural acceptability.

**TABLE 3:** Cost of the baseline and simulated minimum deviation diets for an average adult female.

Main food categories	Baseline Finnish diet in 2017		Health only		Health and GHGE -33%		Health and GHGE -50%	
	€/cap/d	Share	€/cap/d	Share	€/cap/d	Share	€/cap/d	Share
Alcohol	0.38	0.07	0.36	0.07	0.35	0.07	0.25	0.06
Beverages	0.27	0.05	0.26	0.05	0.17	0.04	0.08	0.020
Cereals	0.23	0.04	0.27	0.05	0.28	0.06	0.32	0.08
Diet products	0.07	0.01	0.07	0.01	0.07	0.01	0.08	0.02
Eggs	0.08	0.01	0.09	0.02	0.10	0.02	0.09	0.02
Fats	0.20	0.04	0.20	0.04	0.23	0.05	0.21	0.05
Fish	0.36	0.07	0.49	0.09	0.45	0.09	0.42	0.11
Flavoring	0.03	0.01	0.03	0.01	0.03	0.01	0.03	0.01
Fruits	0.73	0.13	0.81	0.15	0.74	0.15	0.56	0.14
Ingredients	0.04	0.01	0.06	0.01	0.06	0.01	0.07	0.02
Legumes	0.17	0.03	0.20	0.04	0.23	0.05	0.26	0.06
Meat	0.81	0.15	0.59	0.11	0.34	0.07	0.11	0.03
Milk	1.13	0.21	0.82	0.15	0.79	0.17	0.73	0.18
Potatoes	0.07	0.01	0.08	0.02	0.09	0.02	0.13	0.03
Sugars	0.27	0.05	0.21	0.04	0.23	0.05	0.25	0.06
Vegetables	0.62	0.11	0.80	0.15	0.63	0.13	0.43	0.11
Total	5.45	1.00	5.34	1.00	4.78	1.00	4.00	1.00

The main food categories are defined in Table 2.

Abbreviation: GHGE, greenhouse gas emission.

**TABLE 4:** Energy from the baseline and simulated minimum deviation diets for an average adult female.

Main food categories	Baseline Finnish diet in 2017		Health only		Health and GHGE -33%		Health and GHGE -50%	
	kJ/cap/d	Share	kJ/cap/d	Share	kJ/cap/d	Share	kJ/cap/d	Share
Alcohol	129	0.02	122	0.02	118	0.02	84	0.01
Beverages	100	0.01	98	0.01	71	0.01	45	0.006
Cereals	1668	0.23	2052	0.28	2175	0.30	2660	0.36
Diet products	27	0.00	27	0.00	29	0.00	32	0.00
Eggs	135	0.02	157	0.02	173	0.02	146	0.02
Fats	1062	0.15	1098	0.15	1245	0.17	1116	0.15
Fish	174	0.02	243	0.03	223	0.03	208	0.03
Flavoring	22	0.00	22	0.00	22	0.00	23	0.00
Fruits	589	0.08	666	0.09	606	0.08	485	0.07
Ingredients	44	0.01	73	0.01	85	0.01	88	0.01
Legumes	292	0.04	360	0.05	413	0.06	456	0.06
Meat	746	0.10	540	0.07	347	0.05	90	0.01
Milk	1359	0.19	869	0.12	839	0.11	804	0.11
Potatoes	260	0.036	310	0.04	337	0.05	474	0.06
Sugars	506	0.07	425	0.06	425	0.06	428	0.06
Vegetables	198	0.03	250	0.03	202	0.03	158	0.02
Total	7311	1.00	7311	1.00	7311	1.00	7311	1.00

The main food categories are defined in Table 2.

Abbreviation: GHGE, greenhouse gas emission.

**TABLE 5:** GHGE from the baseline and simulated minimum deviation diets for an average adult female.

Main food categories	Baseline Finnish diet in 2017		Health only		Health and GHGE -33%		Health and GHGE -50%	
	kg CO_2_e/cap/d	Share	kg CO_2_e/cap/d	Share	kg CO_2_e/cap/d	Share	kg CO_2_e/cap/d	Share
Alcohol	0.07	0.02	0.06	0.02	0.06	0.02	0.04	0.02
Beverages	0.21	0.05	0.20	0.06	0.12	0.05	0.04	0.02
Cereals	0.17	0.05	0.20	0.06	0.21	0.09	0.26	0.14
Diet products	0.00	0.00	0.00	0.00	0.00	0.00	0.00	0.00
Eggs	0.07	0.02	0.08	0.02	0.09	0.03	0.07	0.04
Fats	0.24	0.06	0.18	0.05	0.21	0.08	0.18	0.10
Fish	0.12	0.03	0.17	0.05	0.15	0.06	0.14	0.08
Flavoring	0.00	0.00	0.00	0.00	0.00	0.00	0.00	0.00
Fruits	0.25	0.07	0.28	0.09	0.25	0.10	0.19	0.10
Ingredients	0.00	0.00	0.00	0.00	0.00	0.00	0.00	0.00
Legumes	0.04	0.01	0.05	0.01	0.05	0.02	0.05	0.03
Meat	1.35	0.36	0.95	0.30	0.52	0.21	0.25	0.13
Milk	0.86	0.23	0.54	0.17	0.50	0.20	0.44	0.24
Potatoes	0.01	0.00	0.01	0.00	0.01	0.00	0.02	0.01
Sugars	0.10	0.03	0.08	0.03	0.09	0.03	0.09	0.05
Vegetables	0.30	0.08	0.39	0.12	0.26	0.10	0.12	0.06
Total	3.78	1.00	3.20	1.00	2.52	1.00	1.89	1.00

The main food categories are defined in Table 2.

Abbreviation: GHGE, greenhouse gas emission.

The composition of the bars in FIGURE 1, FIGURE 2 reveal the main mechanisms driving this result. First, there is a considerable reduction in the consumption of meat, which accounts for 14% (10%) of energy intake in the baseline diet but only 4% (1%) in the health and GHGE -50% scenario for an adult male (female). The reduction in energy intake from dairy products is- less pronounced but also large, particularly for males (17% in baseline compared with 9% in the health and GHGE -50% diets), as well as for alcoholic and nonalcoholic beverages. The loss of energy from those categories is compensated primarily by increases in the consumption of cereals, which end up accounting for a large share of energy intake for the health and GHGE -50% diets (42% for a male and 36% for a female). Potatoes also grow in importance as a source of energy in reduced GHGE diets, particularly for females, but from a smaller base than cereals (i.e., the share of energy rises from 3.6% at the baseline to 6.5% for the health and GHGE -50% diet).

In addition to this broad substitution away from meat and dairy productions toward cereals and potatoes, we note a smaller importance of beverages in nutritionally adequate and reduced GHGE diets. In particular, the energy share of nonalcoholic beverages drops by more than half for females between the baseline and the health and GHGE -50% scenario. Legumes also increase as a source of energy in the diets with lower GHGE. For the remaining categories, including fruits, vegetables, fats, and sugar products, the energy shares do not change substantially from the baseline across the simulated scenarios.

The previous figures and tables hide some adjustments in consumption that occur within the 16 main food categories, keeping in mind that the model is set up in terms of 74 food categories. By applying a simple additive decomposition (Supplemental Material: Mathematical Appendix), Table 6 and Supplemental Table 6 present, for each main food category and an average adult male and an average adult female, respectively, the contribution of those intra-category adjustments to changes in cost and GHGE. The results show that those intracategory contributions are often considerable, although the exact pattern depends on the sex of the individual and variable considered (i.e., cost and GHGE). For instance, for an average male, intra-category substitutions account for 46% of the reduction in GHGE in the health and GHGE -33% scenario (i.e., −0.81 kg/cap/d compared with −0.96 kg/cap/d for inter-category substitutions), mainly due to substitutions within the meat and milk categories away from relatively high-impact categories (e.g., beef) toward lower impact ones (e.g., poultry). Further, the effect of intra-category substitutions on diet cost is consistently negative across scenarios and sexes. This effect reinforces that outlined above centering on the substitution of meat and dairy products with cereal products and potatoes to explain why the low-impact diets are also cheaper than current diets. Thus, the general shift from relatively high-price foods toward more affordable ones occurs both across main food categories and within those.

**TABLE 6:** Additive decomposition of changes in cost and GHGE for an average adult male.

Main food categories	Health only		Health and GHGE - 33%		Health and GHGE - 50%	
	Intra	Inter	Intra	Inter	Intra	Inter
Cost (€/cap/d)						
Alcohol	0.02	−0.16	0.02	−0.16	0.10	−0.41
Beverages	−0.02	−0.01	−0.03	−0.02	−0.09	−0.05
Cereals	0.00	0.15	0.00	0.15	−0.01	0.18
Diet products	0.00	0.00	0.00	0.00	0.00	0.00
Eggs	0.00	−0.01	0.00	−0.01	0.00	0.00
Fats	−0.01	−0.03	−0.01	−0.02	0.00	−0.01
Fish	0.00	0.01	0.00	0.00	0.01	−0.09
Flavoring	0.00	0.00	0.00	0.00	0.00	0.00
Fruits	−0.01	0.06	−0.01	0.05	−0.03	−0.03
Ingredients	0.01	0.00	0.01	0.00	0.00	0.00
Legumes	0.00	0.04	0.00	0.04	0.01	0.03
Meat	−0.04	−0.50	−0.08	−0.57	−0.19	−0.91
Milk	−0.11	−0.40	−0.10	−0.40	−0.17	−0.31
Potatoes	0.00	0.01	0.00	0.01	0.00	0.03
Sugars	0.00	−0.04	0.00	−0.03	0.00	−0.01
Vegetables	−0.02	0.18	−0.02	0.16	−0.01	0.00
Total	−0.18	−0.69	−0.23	−0.79	−0.39	−1.58
GHGE (kg/cap/d)						
Alcohol	0.00	−0.04	0.00	−0.04	0.00	−0.09
Beverages	−0.01	−0.01	−0.02	−0.01	−0.09	−0.03
Cereals	−0.01	0.12	−0.01	0.13	−0.01	0.16
Diet products	0.00	0.00	0.00	0.00	0.00	0.00
Eggs	0.00	−0.01	0.00	0.00	0.00	0.00
Fats	−0.09	−0.02	−0.08	−0.02	−0.08	−0.01
Fish	0.00	0.00	0.00	0.00	0.00	−0.03
Flavoring	0.00	0.00	0.00	0.00	0.00	0.00
Fruits	0.00	0.02	0.00	0.02	−0.01	−0.01
Ingredients	0.00	0.00	0.00	0.00	0.00	0.00
Legumes	0.00	0.01	0.00	0.01	0.00	0.01
Meat	−0.11	−0.81	−0.41	−0.83	−0.56	−1.32
Milk	−0.28	−0.27	−0.28	−0.27	−0.34	−0.20
Potatoes	0.00	0.00	0.00	0.00	0.00	0.00
Sugars	0.00	−0.02	0.00	−0.01	0.00	−0.01
Vegetables	0.00	0.08	−0.01	0.07	−0.03	0.00
Total	−0.51	−0.92	−0.81	−0.96	−1.13	−1.51

“Intra” denotes the effect of intracategory substitutions, “inter” the effect of intercategory substitutions. The main food categories are defined in Table 2.

Abbreviation: GHGE, greenhouse gas emission.

### Are there large differences in diet costs across sociodemographic groups?

**FIGURE 3:**
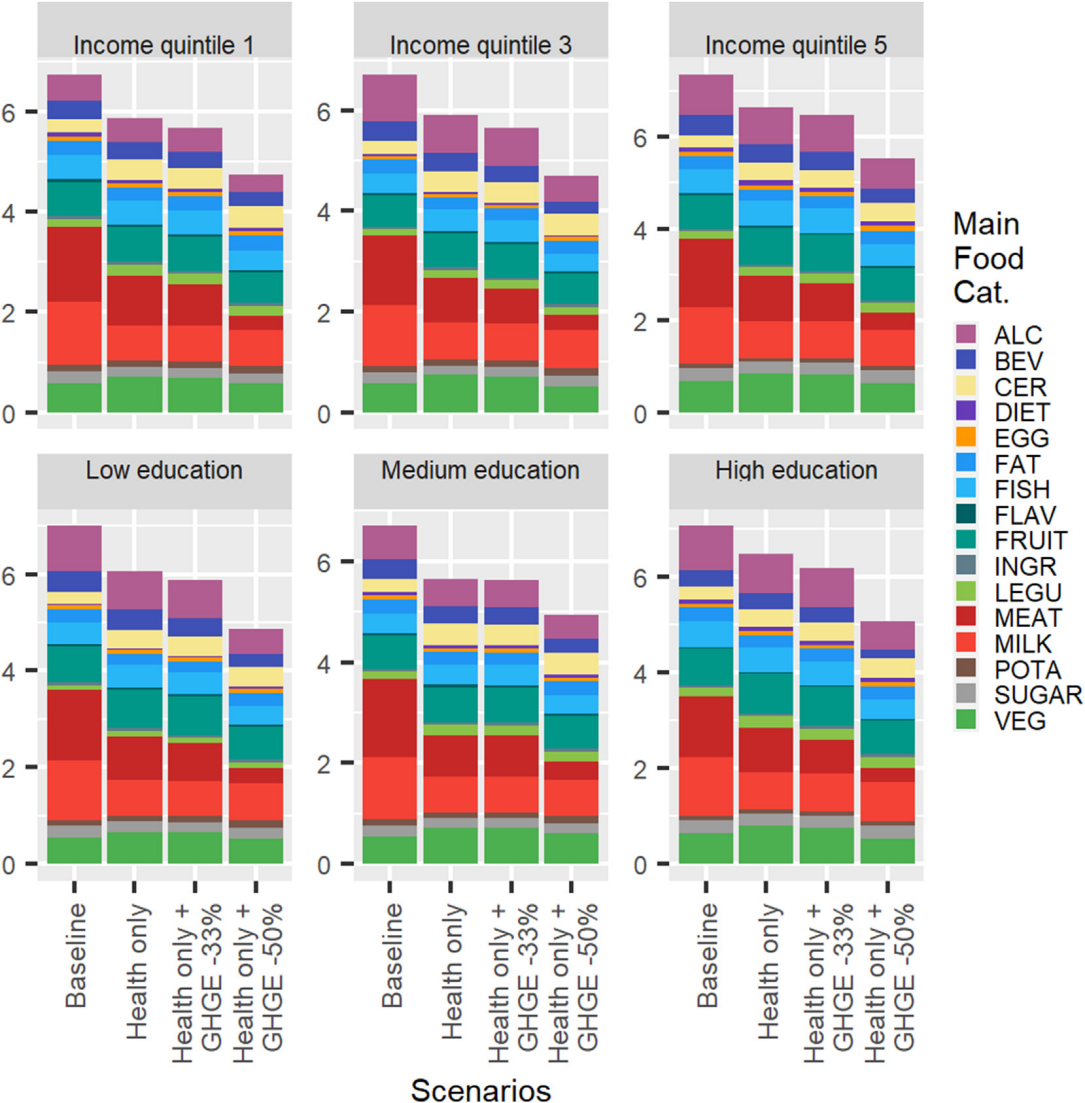
Diet cost (€/cap/d) across sociodemographic groups: males. The main food categories are defined in Table 2.

**FIGURE 4:**
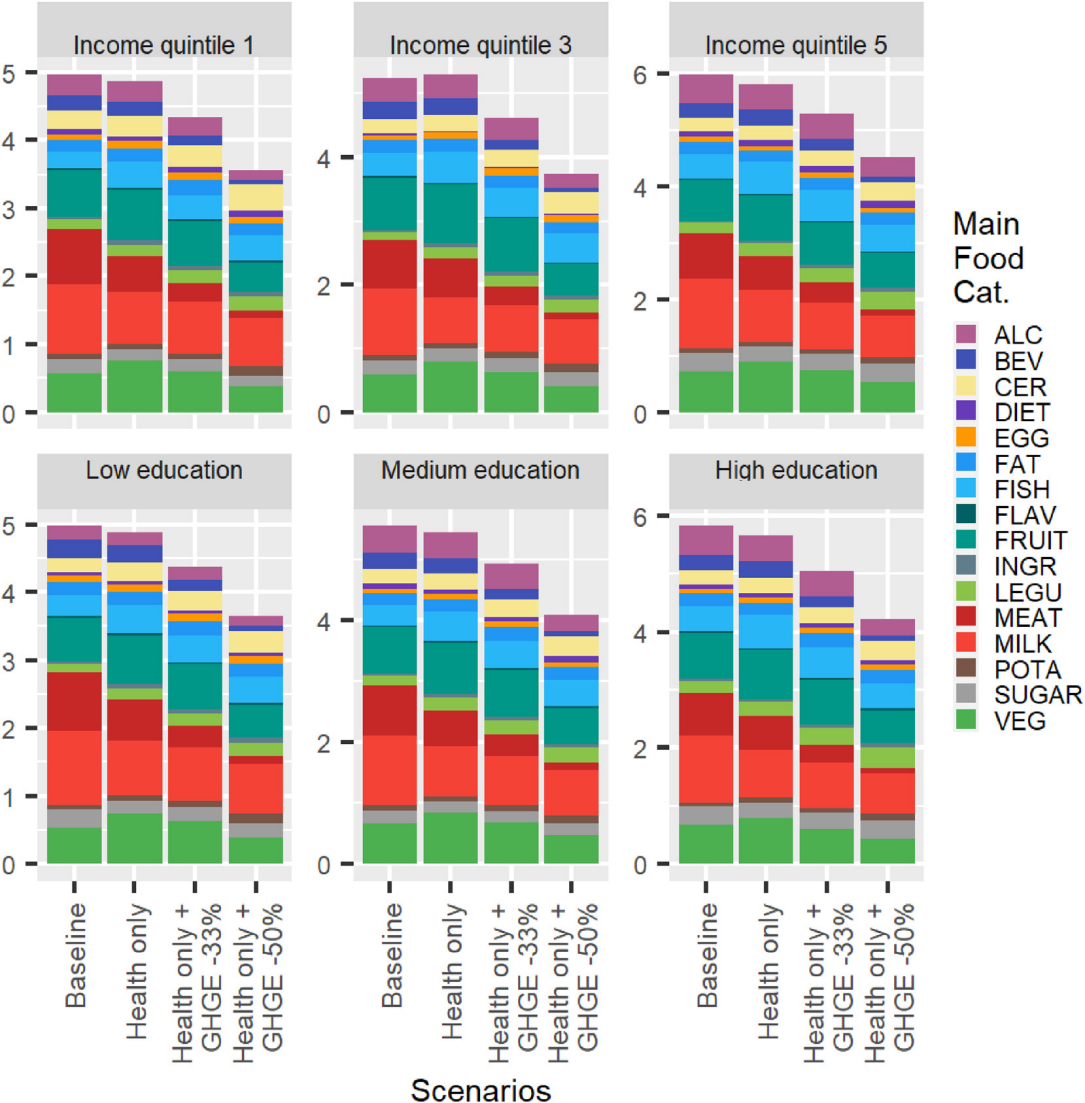
Diet cost (€/cap/d) across sociodemographic groups: females. The main food categories are defined in Table 2.

FIGURE 3, FIGURE 4 display the results of the simulations expressed in terms of diet cost for different sociodemographic groups of the Finnish population, depending on their income or level of education. The pronounced similarity of the bar charts across subpopulations indicates that the key results and mechanisms highlighted earlier for an average female and average male are very robust to differences in sociodemographic characteristics. In particular, health-only diets are always less costly than current diets, and the cost of the simulated minimum deviation diets decreases as the GHGE constraint is imposed and tightened (health and GHGE -33% and health and GHGE -50% scenarios).

In fact, the only difference in diet costs that is readily observed in FIGURE 1, FIGURE 2 is between the 2 sexes. Males spend 24% more on food at the baseline than females, but this is almost entirely explained by higher energy intakes (+28% on average). From that baseline, the reduction in cost to achieve a nutritionally adequate diet is more pronounced for males because they have to make larger adjustments to comply with nutritional requirements. The percentage reductions in diet cost for the health and GHGE -33% and health and GHGE -50% scenarios are, however, very similar for males and females.

## Discussion

Our analysis first demonstrated that relatively low cost and nutritionally adequate diets with a reduced climate impact can be designed from the foods currently supplied by the Finnish food system but that those minimum cost diets severely lack variety. In particular, they rule out the consumption of entire categories of foods (e.g., fruits and processed dairy products) that occupy an important place in current diets. While defining cultural acceptability may be difficult, there is little doubt that the simulated minimum cost diets are not currently acceptable and would not be adopted by Finnish consumers unless extremely coercive and currently politically unacceptable measures (e.g., bans) were introduced.

This lack of diversity and cultural acceptability of minimum cost diets has been established previously in the literature [52]^,^ and studies have shown that the modeling of healthy diets with a low climate impact is sensitive to the consideration of acceptability constraints [53]. Although our result is not unexpected, it is useful in establishing that, in the context of a high-income country such as Finland, the key barrier to the adoption of sustainable diets is not economic but rather cultural, even for relatively low-income households (i.e., belonging to the first income quintile; however, this does exempt from the need to consider impacts of sustainable diet policies on the most vulnerable in society). This contrasts with a strand of the literature that has emphasized the relatively high price of nutritionally rich foods as an important barrier to the adoption of healthy diets [27].

The results of the minimum deviation model simulations reinforce the idea that cultural acceptability is paramount to understanding the barriers to the adoption of sustainable diets. The simulations establish that the lower the maximum permissible level of GHGE from nutritionally adequate diets, the lower their cost. This implies that the budget constraint is not the reason why those nutritionally adequate and reduced GHGE diets are not adopted. It also follows that, for most individuals, the adoption of reduced GHGE diets is a sacrifice that relates exclusively to cultural acceptability, because adoption would generate side benefits in terms of both nutrition adequacy (so presumably health) and reduced cost.

Thus, a general conclusion is that the issues of taste, convenience, compliance with social norms, and other cultural aspects need to receive considerably more attention than in the current literature on sustainable diets. The difficulty lies with the multidimensional, heterogeneous, and somewhat subjective nature of cultural acceptability, which implies the need for multidisciplinary approaches combining natural and social sciences. There is no doubt that our own approach combining minimum deviation from observed diets and food habit constraints presents some limitations—for instance, we could have legitimately used as an alternative objective function of the minimum deviation model a weighted relative square deviation or an absolute deviation (as mentioned in a recent review [39] of diet optimization: “Because no form is better than the others, the choice must be made on the basis of a hypothesis of what the population of interest would find most acceptable.”) There is also no theoretical or empirical reason why a 1% change in the consumption of a given category, such as beef and lamb, is equally difficult for a consumer as a 1% reduction in consumption of any other category, such as apple, but that is implicitly what is implied by the choice of our objective function. However, as noted in a review of the use of linear programming to optimize diets nutritiously, economically, and environmentally, “no study has provided the ultimate solution to calculating acceptability” [54]. Thus, improvements in diet optimization models should focus on developing empirically driven objective functions capable of measuring the difficulty for current consumers of substituting foods for one another or what is known as the taste cost of a dietary adjustment [55]. An early effort [56] based on the economic notion of consumer surplus and calibrated using empirically estimated price elasticities demonstrated the feasibility of the approach. However, the proposed objective function remained simplistic in its treatment of cross-product substitutions, and there is, therefore, much room for improvement of quantitative diet optimization models. There is also a need for qualitative work with consumers and ordinary citizens to complement quantitative analyses in order to understand the real potential for and cultural obstacles to the adoption of sustainable diets.

In addition to the problems linked to the measurement of cultural acceptability, our research features some limitations that must be acknowledged. An important one relates to the fact that the model is built on food ingredient categories rather than the original food categories (i.e., foods as eaten) recorded in the dietary intake survey. This creates a difficulty in measuring costs and affects slightly the climate coefficients. By valuing only the ingredients rather than the final foods themselves, we are likely to underestimate diet costs and the climate impact. However, as long as this bias is systematic and applies to both current diets and simulated diets, it should not distort the results expressed in relative (i.e., percentage) terms. Nevertheless, there is a crucial need to refine and harmonize food classifications across the nutritional, environmental and economic domains to support a comprehensive analysis of sustainable diets in the future.

From the point of view of consumers, the finding that nutritionally adequate, reduced GHGE diets cost less than current ones is of course positive. The analysis also reveals that this conclusion holds across sociodemographic groups defined in terms of education and income level, as the composition of the baseline diets and nature of the simulated dietary adjustments remain very similar in the subpopulations that we have analyzed. This implies that the policies promoting the adoption of nutritionally adequate and climate-friendly diets are likely to be progressive (we acknowledge, however, that the exact impact will depend on the chosen policy instruments - for example, taxes compared with informational campaign - to drive behavioral change): all consumers benefit from lower diet costs by roughly the same absolute amount, but low-income groups benefit relatively more due to Engle's law (i.e., the share of food in their budget is larger).

However, when considering the entire food system, one must also acknowledge that the decrease in the volume of food consumption overall in the simulated scenarios represents a net reduction in demand for food suppliers, which would reduce value added in food production, processing and distribution [57]. Further, that reduction would not be spread uniformly across the subsectors of the food industry, with particularly large impacts on the sectors that form the bulk of the Finnish agrifood system, namely dairy and meat. Those impacts, and how to reduce them, need to be analyzed in an attempt to minimize them and, by so doing, remove key political obstacles to the adoption of sustainable diet policies. Nevertheless, we believe that our analysis provides evidence that will help design realistic pathways for the just transition of the Finnish food system, in conjunction with changes affecting land use, agricultural practices, agrifood technology, or reductions in food waste [58].

Finally, while confirming the general direction of dietary adjustments for sustainability, our results suggest that the message sent to Finnish consumers should be more nuanced than just “eat more plants, less animals.” Reductions in meat consumption are the key to lower climate impact, but dairy products still account for a large share of energy in the simulated reduced GHGE diets, whereas consumption of fruits and vegetables remains at levels close to their currently observed ones (and substantially lower in the health and GHGE -50% scenario). The latter result reflects the relatively large climate impact of fruits and vegetables once expressed on a per-calorie basis, which implies that isocaloric substitutions of animal products with fruits and vegetables may not be desirable from a climate point of view, as reported elsewhere [59]. However, because low fruit and vegetable consumption is a major risk factor for many noncommunicable diseases [60], a decrease is not recommendable, but rather intra-category substitutions to more local and seasonal fruits and vegetables with lower carbon footprints should be investigated further. In this research, the health aspect was taken into account only via adequacy of nutrients but, in future optimization studies, health should be considered as a broader construct.

At another level, significant reductions in GHGE can also be achieved by decreasing consumption of alcoholic and nonalcoholic beverages, categories that are often forgotten in the discussion of sustainable food systems. Finally, our findings suggest that more emphasis should be placed on intra-category substitutions because those can deliver large climate benefits and are likely to be less difficult to achieve by consumers. This idea should, however, be analyzed while considering potential trade-offs with other environmental impacts, such as within the meat category, where broiler meat has a relatively large impact on biodiversity (measured as the potentially disappeared fraction of species on a global level) compared with beef [16].

## Acknowledgments

We thank colleagues of the Just Food project for providing insightful comments and constructive feedback during project meetings.

### Authors contributions

The authors’ responsibilities were as follows – all authors: designed the study; XI: coded the models, ran simulations, analyzed the results, wrote a complete first draft, led the revisions, and had primary responsibility for the final content; JN: contributed to the formulation of the model, commented and edited the manuscript; LP, LS-J: contributed to the formulation of the model, data preparation and analysis of the results, commented on and edited the manuscript; MS: prepared the environmental data, contributed to the formulation of the model, commented on and edited the manuscript; HT: prepared the food intake and nutritional data, contributed to the formulation of the model, commented on and edited the manuscript; LV: contributed to the formulation of the model and data preparation, commented on and edited the manuscript; and all authors: read and approved the submitted manuscript.

## Conflict of Interest

The authors report no conflicts of interest.

## Funding

This work was funded by the Strategic Research Council (SRC) established within the Research Council of Finland, project Just Food “Just transition: tackling inequalities on the way to a sustainable, healthy and climate neutral food system,” grant numbers 352640, 352641, and 352638. The dietary data collection was also partially funded by the European Food Safety Authority (EFSA), contract OC/EFSA/DATA/2015/03 CT 01 (EU Menu, Lot 2, Finland/Adults). The publication is produced by the authors and their organizations and not by EFSA and only represents the views of the authoring parties and not EFSA’s position.

## Data availability

The R codes of the 2 diet optimization models are available from the corresponding author on request. Data described in the manuscript will not be made available because of the conditions imposed by the contracts that the authors signed to gain access to the FinDiet data and HBS data. For research purposes, the HBS data can be purchased directly from Statistics Finland subject to specific conditions. The data presented in this study (the FinDiet 2017 Survey data) are available on request from THL Biobank at: https://thl.fi/en/web/thl-biobank/for-researchers (accessed on 11 October, 2023). The individual-level data are not publicly available owing to privacy restrictions.
