# Supplementary material for: Assessing the Cost of Nutritionally Adequate and Low-Climate Impact Diets in Finland
Source: Curr Dev Nutr. 2024 Apr 3;8(5):102151. doi: 10.1016/j.cdnut.2024.102151 (PMC11090877; doi:10.1016/j.cdnut.2024.102151)
Supplement: Multimedia component 1 [file mmc1.docx]

**Mathematical appendix – Decomposition of total changes in intra-category and inter-category substitutions:**

Suppose that we are concerned with an indicator (e.g., GHGE, cost, energy) *D* which has a constant coefficient *a_i_* for each food category *i* consumed in quantity *x_i_*. The issue is to decompose *∆D* between the baseline and any scenario: $\Delta D=\sum_{i=1}^{n} a_{i}{(x_{i}^{'}-x}_{i})=\sum_{i=1}^{n} a_{i}\Delta x_{i}$, where $x_{i}$ denotes consumption at the baseline and $x_{i}^{'}$ denotes consumption in the considered scenario.

We define for any main food category G the aggregate quantity $x_{G}=\sum_{j=1}^{n_{G}} x_{j}$ , where *n_G_* denotes the number of food categories in the main food category G, and the aggregate coefficient $a_{G}=\sum_{j=1}^{n_{G}} a_{j}x_{j}/x_{G}$. It follows that $\Delta D=a_{G}^{'}x_{G}^{'}-a_{G}x_{G}=a_{G}^{'}x_{G}^{'}-a_{G}^{'}x_{G}+a_{G}^{'}x_{G}-a_{G}x_{G}=a_{G}^{'}\left( x_{G}^{'}-x_{G} \right)+x_{G}\left( a_{G}^{'}-a_{G} \right)=a_{G}^{'}\Delta x_{G}+x_{G}\Delta a_{G}$. The first term measures the impact of inter-category substitutions on the change in indicator, the second term measures the impact of inter-category substitutions.
