# Supplementary material for: Assessing the Cost of Nutritionally Adequate and Low-Climate Impact Diets in Finland
Source: Curr Dev Nutr. 2024 Apr 3;8(5):102151. doi: 10.1016/j.cdnut.2024.102151 (PMC11090877; doi:10.1016/j.cdnut.2024.102151)
Supplement: Multimedia component 2 [file mmc2.docx]

**Table 1.** Nutrient recommendations imposed in the optimization. All quantities are expressed per person per day.

.
