# Supplementary material for: Assessing the Cost of Nutritionally Adequate and Low-Climate Impact Diets in Finland
Source: Curr Dev Nutr. 2024 Apr 3;8(5):102151. doi: 10.1016/j.cdnut.2024.102151 (PMC11090877; doi:10.1016/j.cdnut.2024.102151)
Supplement: Multimedia component 3 [file mmc3.docx]

**Table 2:** The minimum cost diet for an average adult female

| **Main Food category** | | **Quantity (g/cap/day)** | | **GHGE (gCO2e/cap/day)** | | **Cost (€/cap/day)** | |
| --- | --- | --- | --- | --- | --- | --- | --- |
|  | **Food category** | Current diet | Minimum Cost Diet | Current diet | Minimum Cost Diet | Current diet | Minimum Cost Diet |
| **ALCOHOL** | | **55.8** | **0.0** | **0.1** | **0.0** | **0.38** | **0.00** |
|  | Beer and cider | 40.0 | 0.0 | 0.0 | 0.0 | 0.19 | 0.00 |
|  | Wine & spirit | 15.8 | 0.0 | 0.0 | 0.0 | 0.19 | 0.00 |
| **BEVERAGES** | | **581.5** | **0.0** | **0.2** | **0.0** | **0.27** | **0.00** |
|  | Coffee | 384.9 | 0.0 | 0.2 | 0.0 | 0.17 | 0.00 |
|  | Soft drink | 51.1 | 0.0 | 0.0 | 0.0 | 0.08 | 0.00 |
|  | Tea | 145.5 | 0.0 | 0.0 | 0.0 | 0.01 | 0.00 |
| **CEREALS** | | **125.0** | **277.4** | **0.2** | **0.4** | **0.23** | **0.30** |
|  | Barley and barley products | 0.9 | 0.0 | 0.0 | 0.0 | 0.00 | 0.00 |
|  | Cereal/seed/soy drinks | 13.8 | 0.0 | 0.0 | 0.0 | 0.06 | 0.00 |
|  | Other grains | 2.8 | 0.0 | 0.0 | 0.0 | 0.01 | 0.00 |
|  | Oat & oat products | 16.4 | 3.3 | 0.0 | 0.0 | 0.04 | 0.01 |
|  | Rice | 9.1 | 0.0 | 0.0 | 0.0 | 0.02 | 0.00 |
|  | Rye | 26.5 | 60.4 | 0.0 | 0.1 | 0.05 | 0.12 |
|  | Starches | 2.3 | 0.0 | 0.0 | 0.0 | 0.01 | 0.00 |
|  | Wheat | 53.1 | 213.7 | 0.1 | 0.3 | 0.04 | 0.17 |
| **DIET PRODUUCTS** | | **1.9** | **0.0** | **0.0** | **0.0** | **0.07** | **0.00** |
|  | Sport foods, nutri. support | 1.9 | 0.0 | 0.0 | 0.0 | 0.07 | 0.00 |
| **EGGS** | | **24.1** | **0.0** | **0.1** | **0.0** | **0.08** | **0.00** |
|  | Eggs | 24.1 | 0.0 | 0.1 | 0.0 | 0.08 | 0.00 |
| **FATS** | | **38.3** | **36.8** | **0.2** | **0.1** | **0.20** | **0.18** |
|  | Butter | 3.5 | 0.0 | 0.1 | 0.0 | 0.02 | 0.00 |
|  | Blended spread | 9.7 | 0.0 | 0.1 | 0.0 | 0.06 | 0.00 |
|  | Cooking & animal fat | 4.8 | 0.0 | 0.0 | 0.0 | 0.02 | 0.00 |
|  | Oils | 8.7 | 17.7 | 0.0 | 0.1 | 0.05 | 0.10 |
|  | Salad dressings | 3.1 | 0.0 | 0.0 | 0.0 | 0.02 | 0.00 |
|  | Margarines >= 55% | 5.8 | 19.0 | 0.0 | 0.1 | 0.02 | 0.08 |
|  | Margarines < 55% | 2.7 | 0.0 | 0.0 | 0.0 | 0.01 | 0.00 |
| **FISH** | | **27.8** | **19.4** | **0.1** | **0.1** | **0.36** | **0.20** |
|  | Fish, seafood | 19.2 | 19.4 | 0.1 | 0.1 | 0.20 | 0.20 |
|  | Fish products | 8.6 | 0.0 | 0.0 | 0.0 | 0.15 | 0.00 |
| **FLAVOURING** | | **6.2** | **0.0** | **0.0** | **0.0** | **0.03** | **0.00** |
|  | Condiments | 6.1 | 0.0 | 0.0 | 0.0 | 0.03 | 0.00 |
|  | Dried spices and herbs | 0.1 | 0.0 | 0.0 | 0.0 | 0.00 | 0.00 |
| **FRUITS** | | **279.0** | **0.0** | **0.2** | **0.0** | **0.73** | **0.00** |
|  | Malaceous fruit | 48.5 | 0.0 | 0.0 | 0.0 | 0.09 | 0.00 |
|  | Berries | 34.6 | 0.0 | 0.0 | 0.0 | 0.14 | 0.00 |
|  | Citrus fruit | 32.2 | 0.0 | 0.0 | 0.0 | 0.06 | 0.00 |
|  | Juice drink | 49.3 | 0.0 | 0.1 | 0.0 | 0.21 | 0.00 |
|  | Canned fruit | 5.1 | 0.0 | 0.0 | 0.0 | 0.02 | 0.00 |
|  | Other fruits | 68.9 | 0.0 | 0.1 | 0.0 | 0.16 | 0.00 |
|  | Juices, incl. vegetable juices | 40.4 | 0.0 | 0.0 | 0.0 | 0.06 | 0.00 |
| **INGREDIENTS** | | **7.8** | **5.4** | **0.0** | **0.0** | **0.04** | **0.03** |
|  | Misc. Ingredients | 4.7 | 3.7 | 0.0 | 0.0 | 0.03 | 0.02 |
|  | Salt | 3.1 | 1.8 | 0.0 | 0.0 | 0.01 | 0.00 |
|  | Sweeteners | 0.0 | 0.0 | 0.0 | 0.0 | 0.00 | 0.00 |
| **LEGUMES & NUTS** | | **21.9** | **0.0** | **0.0** | **0.0** | **0.17** | **0.00** |
|  | Nuts & seeds | 8.6 | 0.0 | 0.0 | 0.0 | 0.12 | 0.00 |
|  | Pulse vegetables & products | 10.6 | 0.0 | 0.0 | 0.0 | 0.04 | 0.00 |
|  | Soya products | 2.7 | 0.0 | 0.0 | 0.0 | 0.01 | 0.00 |
| **MEAT** | | **107.1** | **12.6** | **1.4** | **0.2** | **0.81** | **0.10** |
|  | Beef and lamb/mutton | 19.7 | 0.0 | 0.6 | 0.0 | 0.20 | 0.00 |
|  | Cold cuts, meat prod. | 13.6 | 0.0 | 0.2 | 0.0 | 0.15 | 0.00 |
|  | Offal | 1.8 | 12.6 | 0.0 | 0.2 | 0.01 | 0.10 |
|  | Pork and game | 20.4 | 0.0 | 0.1 | 0.0 | 0.17 | 0.00 |
|  | Poultry | 36.1 | 0.0 | 0.2 | 0.0 | 0.17 | 0.00 |
|  | Sausages | 13.3 | 0.0 | 0.1 | 0.0 | 0.08 | 0.00 |
|  | Cold cuts, sausages | 2.2 | 0.0 | 0.0 | 0.0 | 0.02 | 0.00 |
| **DAIRY** | | **395.1** | **532.9** | **0.9** | **0.5** | **1.13** | **0.50** |
|  | Ripened/processed cheese >17% | 17.2 | 0.0 | 0.2 | 0.0 | 0.15 | 0.00 |
|  | Ripened/processed cheese <=17% | 5.1 | 0.0 | 0.1 | 0.0 | 0.05 | 0.00 |
|  | Unripened/fresh cheese > 15% | 6.0 | 0.0 | 0.0 | 0.0 | 0.05 | 0.00 |
|  | Unripened/fresh cheese <= 15% | 9.1 | 0.0 | 0.1 | 0.0 | 0.08 | 0.00 |
|  | Cream | 14.3 | 0.0 | 0.0 | 0.0 | 0.07 | 0.00 |
|  | Quark | 27.0 | 0.0 | 0.0 | 0.0 | 0.23 | 0.00 |
|  | Ice-cream | 9.2 | 0.0 | 0.0 | 0.0 | 0.05 | 0.00 |
|  | Skimmed milk | 77.0 | 243.3 | 0.1 | 0.2 | 0.08 | 0.26 |
|  | Milks > 2% fat | 12.7 | 289.6 | 0.0 | 0.3 | 0.01 | 0.24 |
|  | Milk powders | 0.6 | 0.0 | 0.0 | 0.0 | 0.01 | 0.00 |
|  | Milks <= 2% fat | 116.9 | 0.0 | 0.1 | 0.0 | 0.13 | 0.00 |
|  | Soured/cultured milk | 38.9 | 0.0 | 0.0 | 0.0 | 0.06 | 0.00 |
|  | Fermented milk products, other | 4.9 | 0.0 | 0.0 | 0.0 | 0.02 | 0.00 |
|  | Yoghurt | 56.2 | 0.0 | 0.1 | 0.0 | 0.13 | 0.00 |
| **POTATOES** | | **61.9** | **218.4** | **0.0** | **0.0** | **0.07** | **0.18** |
|  | Potato products | 6.4 | 0.0 | 0.0 | 0.0 | 0.02 | 0.00 |
|  | Potato | 55.4 | 218.4 | 0.0 | 0.0 | 0.05 | 0.18 |
| **SUGAR** | | **32.5** | **0.0** | **0.1** | **0.0** | **0.27** | **0.00** |
|  | Chocolate | 7.2 | 0.0 | 0.0 | 0.0 | 0.11 | 0.00 |
|  | Jam | 4.8 | 0.0 | 0.0 | 0.0 | 0.03 | 0.00 |
|  | Sugar and syrups | 12.8 | 0.0 | 0.0 | 0.0 | 0.03 | 0.00 |
|  | Non-chocolate confectionery | 7.7 | 0.0 | 0.0 | 0.0 | 0.11 | 0.00 |
| **VEGETABLES** | | **192.1** | **96.4** | **0.3** | **0.0** | **0.62** | **0.22** |
|  | Cabbage | 15.2 | 96.4 | 0.0 | 0.0 | 0.04 | 0.22 |
|  | Edible fungi | 2.8 | 0.0 | 0.0 | 0.0 | 0.01 | 0.00 |
|  | Root vegetables and tubers | 28.9 | 0.0 | 0.0 | 0.0 | 0.06 | 0.00 |
|  | Canned vegetables | 13.9 | 0.0 | 0.0 | 0.0 | 0.06 | 0.00 |
|  | Fruit vegetables | 95.2 | 0.0 | 0.2 | 0.0 | 0.25 | 0.00 |
|  | Leaf vegetables | 24.0 | 0.0 | 0.1 | 0.0 | 0.18 | 0.00 |
|  | Onion-family vegetables | 12.2 | 0.0 | 0.0 | 0.0 | 0.02 | 0.00 |
| **ALL** | | **1957.9** | **1199.2** | **3780.0** | **1332.0** | **5.45** | **1.71** |
