# Supplementary material for: Assessing the Cost of Nutritionally Adequate and Low-Climate Impact Diets in Finland
Source: Curr Dev Nutr. 2024 Apr 3;8(5):102151. doi: 10.1016/j.cdnut.2024.102151 (PMC11090877; doi:10.1016/j.cdnut.2024.102151)
Supplement: Multimedia component 4 [file mmc4.docx]

**Table 3:** Cost of the baseline and simulated minimum deviation, average adult male. The main food categories are defined in Table 2.

|  | **Baseline Finnish diet in 2017** | | **Health only** | | **Health &**  **GHGE -33%** | | **Health & GHGE -50%** | |
| --- | --- | --- | --- | --- | --- | --- | --- | --- |
| **Main Food Categories** | €/cap/day | Share | €/cap/day | Share | €/cap/day | Share | €/cap/day | Share |
| **Alcohol** | 0.83 | 0.12 | 0.70 | 0.12 | 0.69 | 0.12 | 0.52 | 0.10 |
| **Beverages** | 0.40 | 0.06 | 0.36 | 0.06 | 0.36 | 0.06 | 0.26 | 0.05 |
| **Cereals** | 0.25 | 0.04 | 0.40 | 0.07 | 0.40 | 0.07 | 0.41 | 0.08 |
| **Diet products** | 0.06 | 0.01 | 0.06 | 0.01 | 0.06 | 0.01 | 0.06 | 0.01 |
| **Eggs** | 0.08 | 0.01 | 0.08 | 0.01 | 0.08 | 0.01 | 0.09 | 0.02 |
| **Fats** | 0.28 | 0.04 | 0.25 | 0.04 | 0.25 | 0.04 | 0.26 | 0.05 |
| **Fish** | 0.45 | 0.07 | 0.46 | 0.08 | 0.45 | 0.08 | 0.38 | 0.08 |
| **Flavouring** | 0.04 | 0.01 | 0.04 | 0.01 | 0.04 | 0.01 | 0.04 | 0.01 |
| **Fruits** | 0.73 | 0.11 | 0.78 | 0.13 | 0.77 | 0.13 | 0.67 | 0.14 |
| **Ingredients** | 0.05 | 0.01 | 0.05 | 0.01 | 0.05 | 0.01 | 0.06 | 0.01 |
| **Legumes** | 0.14 | 0.02 | 0.18 | 0.03 | 0.18 | 0.03 | 0.18 | 0.04 |
| **Meat** | 1.44 | 0.21 | 0.90 | 0.15 | 0.78 | 0.13 | 0.34 | 0.07 |
| **Milk** | 1.23 | 0.18 | 0.72 | 0.12 | 0.72 | 0.12 | 0.75 | 0.15 |
| **Potatoes** | 0.11 | 0.02 | 0.12 | 0.02 | 0.12 | 0.02 | 0.13 | 0.03 |
| **Sugars** | 0.24 | 0.04 | 0.21 | 0.03 | 0.21 | 0.04 | 0.23 | 0.05 |
| **Vegetables** | 0.57 | 0.08 | 0.73 | 0.12 | 0.71 | 0.12 | 0.56 | 0.11 |
| **TOTAL** | **6.90** | **1.00** | **6.03** | **1.00** | **5.88** | **1.00** | **4.94** | **1.00** |
