# Supplementary material for: Assessing the Cost of Nutritionally Adequate and Low-Climate Impact Diets in Finland
Source: Curr Dev Nutr. 2024 Apr 3;8(5):102151. doi: 10.1016/j.cdnut.2024.102151 (PMC11090877; doi:10.1016/j.cdnut.2024.102151)
Supplement: Multimedia component 5 [file mmc5.docx]

**Table 4:** Energy from the baseline and simulated minimum deviation diets, average adult male. The main food categories are defined in Table 2.

|  | **Baseline Finnish diet in 2017** | | **Health only** | | **Health &**  **GHGE -33%** | | **Health & GHGE -50%** | |
| --- | --- | --- | --- | --- | --- | --- | --- | --- |
| **Main Food Categories** | **kJ/cap/day** | **Share** | **kJ/cap/day** | **Share** | **kJ/cap/day** | **Share** | **kJ/cap/day** | **Share** |
| **Alcohol** | 295 | 0.03 | 245 | 0.03 | 244 | 0.03 | 178 | 0.02 |
| **Beverages** | 164 | 0.02 | 149 | 0.02 | 147 | 0.02 | 123 | 0.01 |
| **Cereals** | 2206 | 0.24 | 3559 | 0.38 | 3611 | 0.39 | 3944 | 0.42 |
| **Diet products** | 24 | 0.00 | 25 | 0.00 | 25 | 0.00 | 26 | 0.00 |
| **Eggs** | 136 | 0.01 | 125 | 0.01 | 126 | 0.01 | 142 | 0.02 |
| **Fats** | 1417 | 0.15 | 1322 | 0.14 | 1347 | 0.14 | 1419 | 0.15 |
| **Fish** | 222 | 0.02 | 228 | 0.02 | 223 | 0.02 | 183 | 0.02 |
| **Flavouring** | 33 | 0.00 | 34 | 0.00 | 34 | 0.00 | 35 | 0.00 |
| **Fruits** | 522 | 0.06 | 583 | 0.06 | 577 | 0.06 | 515 | 0.05 |
| **Ingredients** | 62 | 0.01 | 71 | 0.01 | 70 | 0.01 | 78 | 0.01 |
| **Legumes** | 240 | 0.03 | 318 | 0.03 | 316 | 0.03 | 308 | 0.03 |
| **Meat** | 1333 | 0.14 | 814 | 0.09 | 735 | 0.08 | 391 | 0.04 |
| **Milk** | 1625 | 0.17 | 816 | 0.09 | 825 | 0.09 | 879 | 0.09 |
| **Potatoes** | 396 | 0.04 | 419 | 0.04 | 424 | 0.05 | 479 | 0.05 |
| **Sugars** | 499 | 0.05 | 405 | 0.04 | 415 | 0.04 | 464 | 0.05 |
| **Vegetables** | 188 | 0.02 | 249 | 0.03 | 244 | 0.03 | 199 | 0.02 |
| **TOTAL** | **9363** | **1.00** | **9363** | **1.00** | **9363** | **1.00** | **9363** | **1.00** |
