# Supplementary material for: Assessing the Cost of Nutritionally Adequate and Low-Climate Impact Diets in Finland
Source: Curr Dev Nutr. 2024 Apr 3;8(5):102151. doi: 10.1016/j.cdnut.2024.102151 (PMC11090877; doi:10.1016/j.cdnut.2024.102151)
Supplement: Multimedia component 6 [file mmc6.docx]

**Table 5:** GHGE of the baseline and simulated minimum deviation diets, average adult male. The main food categories are defined in Table 2.

|  | **Baseline Finnish diet in 2017** | | **Health only** | | **Health &**  **GHGE -33%** | | **Health & GHGE -50%** | |
| --- | --- | --- | --- | --- | --- | --- | --- | --- |
| **Main Food Categories** | **kg CO2e/ cap/day** | **Share** | **kg CO2e/ cap/day** | **Share** | **kg CO2e/ cap/day** | **Share** | **kg CO2e/ cap/day** | **Share** |
| **Alcohol** | 0.20 | 0.04 | 0.16 | 0.04 | 0.16 | 0.04 | 0.11 | 0.04 |
| **Beverages** | 0.25 | 0.05 | 0.23 | 0.06 | 0.22 | 0.06 | 0.13 | 0.05 |
| **Cereals** | 0.22 | 0.04 | 0.33 | 0.09 | 0.34 | 0.10 | 0.37 | 0.14 |
| **Diet products** | 0.00 | 0.00 | 0.00 | 0.00 | 0.00 | 0.00 | 0.00 | 0.00 |
| **Eggs** | 0.07 | 0.01 | 0.06 | 0.02 | 0.06 | 0.02 | 0.07 | 0.03 |
| **Fats** | 0.33 | 0.06 | 0.22 | 0.06 | 0.22 | 0.06 | 0.24 | 0.09 |
| **Fish** | 0.15 | 0.03 | 0.15 | 0.04 | 0.15 | 0.04 | 0.12 | 0.05 |
| **Flavouring** | 0.01 | 0.00 | 0.01 | 0.00 | 0.01 | 0.00 | 0.01 | 0.00 |
| **Fruits** | 0.25 | 0.05 | 0.26 | 0.07 | 0.26 | 0.07 | 0.23 | 0.09 |
| **Ingredients** | 0.00 | 0.00 | 0.00 | 0.00 | 0.00 | 0.00 | 0.00 | 0.00 |
| **Legumes** | 0.03 | 0.01 | 0.04 | 0.01 | 0.04 | 0.01 | 0.04 | 0.02 |
| **Meat** | 2.38 | 0.45 | 1.47 | 0.38 | 1.14 | 0.32 | 0.50 | 0.19 |
| **Milk** | 1.04 | 0.20 | 0.49 | 0.13 | 0.49 | 0.14 | 0.50 | 0.19 |
| **Potatoes** | 0.01 | 0.00 | 0.01 | 0.00 | 0.01 | 0.00 | 0.02 | 0.01 |
| **Sugars** | 0.10 | 0.02 | 0.08 | 0.02 | 0.08 | 0.02 | 0.09 | 0.04 |
| **Vegetables** | 0.27 | 0.05 | 0.35 | 0.09 | 0.33 | 0.09 | 0.23 | 0.09 |
| **TOTAL** | **5.30** | **1.00** | **3.87** | **1.00** | **3.53** | **1.00** | **2.65** | **1.00** |
