# Supplementary material for: Assessing the Cost of Nutritionally Adequate and Low-Climate Impact Diets in Finland
Source: Curr Dev Nutr. 2024 Apr 3;8(5):102151. doi: 10.1016/j.cdnut.2024.102151 (PMC11090877; doi:10.1016/j.cdnut.2024.102151)
Supplement: Multimedia component 7 [file mmc7.docx]

**Table 6:** Additive decomposition of changes in cost and GHGE, average adult female. “Intra” denotes the effect of intra-category substitutions, “inter” the effect of inter-category substitutions. The main food categories are defined in Table 1.

|  |  | **Health only** | | **Health &**  **GHGE -33%** | | **Health & GHGE -50%** | |
| --- | --- | --- | --- | --- | --- | --- | --- |
|  | **Main Food Categories** | Intra | Inter | Intra | Inter | Intra | Inter |
| **Cost (€/cap/day)** | |  |  |  |  |  |  |
|  | Alcohol | -0.01 | -0.02 | 0.00 | -0.04 | 0.05 | -0.18 |
|  | Beverages | -0.01 | 0.00 | -0.09 | -0.01 | -0.20 | 0.01 |
|  | Cereals | -0.01 | 0.05 | -0.01 | 0.06 | -0.02 | 0.12 |
|  | Diet products | 0.00 | 0.00 | 0.00 | 0.00 | 0.00 | 0.01 |
|  | Eggs | 0.00 | 0.01 | 0.00 | 0.02 | 0.00 | 0.01 |
|  | Fats | 0.00 | 0.00 | 0.00 | 0.03 | 0.00 | 0.01 |
|  | Fish | -0.01 | 0.15 | -0.01 | 0.10 | 0.00 | 0.07 |
|  | Flavouring | 0.00 | 0.00 | 0.00 | 0.00 | 0.00 | 0.00 |
|  | Fruits | -0.01 | 0.09 | -0.02 | 0.02 | -0.05 | -0.12 |
|  | Ingredients | 0.00 | 0.02 | 0.00 | 0.02 | 0.00 | 0.03 |
|  | Legumes | 0.00 | 0.04 | 0.01 | 0.06 | 0.02 | 0.07 |
|  | Meat | -0.03 | -0.19 | -0.05 | -0.42 | 0.20 | -0.91 |
|  | Milk | -0.23 | -0.08 | -0.20 | -0.14 | -0.32 | -0.09 |
|  | Potatoes | 0.00 | 0.02 | 0.00 | 0.03 | -0.01 | 0.06 |
|  | Sugars | -0.03 | -0.03 | -0.01 | -0.03 | 0.02 | -0.04 |
|  | Vegetables | 0.00 | 0.17 | 0.03 | -0.03 | 0.04 | -0.23 |
|  | TOTAL | **-0.32** | **0.22** | **-0.34** | **-0.33** | **-0.27** | **-1.17** |
| **GHGE (kg/cap/day)** | |  |  |  |  |  |  |
|  | Alcohol | 0.00 | 0.00 | 0.00 | -0.01 | 0.00 | -0.03 |
|  | Beverages | -0.01 | 0.00 | -0.08 | -0.01 | -0.17 | 0.00 |
|  | Cereals | 0.00 | 0.03 | 0.00 | 0.05 | -0.01 | 0.09 |
|  | Diet products | 0.00 | 0.00 | 0.00 | 0.00 | 0.00 | 0.00 |
|  | Eggs | 0.00 | 0.01 | 0.00 | 0.02 | 0.00 | 0.01 |
|  | Fats | -0.06 | 0.00 | -0.06 | 0.02 | -0.07 | 0.01 |
|  | Fish | 0.00 | 0.05 | 0.00 | 0.04 | 0.00 | 0.02 |
|  | Flavouring | 0.00 | 0.00 | 0.00 | 0.00 | 0.00 | 0.00 |
|  | Fruits | 0.00 | 0.03 | 0.00 | 0.01 | -0.02 | -0.04 |
|  | Ingredients | 0.00 | 0.00 | 0.00 | 0.00 | 0.00 | 0.00 |
|  | Legumes | 0.00 | 0.01 | 0.00 | 0.01 | 0.00 | 0.01 |
|  | Meat | -0.09 | -0.31 | -0.19 | -0.64 | 1.08 | -2.18 |
|  | Milk | -0.27 | -0.05 | -0.27 | -0.09 | -0.36 | -0.05 |
|  | Potatoes | 0.00 | 0.00 | 0.00 | 0.00 | 0.00 | 0.01 |
|  | Sugars | -0.01 | -0.01 | 0.00 | -0.01 | 0.00 | -0.01 |
|  | Vegetables | 0.01 | 0.09 | -0.03 | -0.01 | -0.12 | -0.06 |
|  | TOTAL | -0.43 | -0.15 | -0.64 | -0.62 | 0.33 | -2.22 |
